# Feasibility of combination of Gun-Chil-Jung and cytokine-induced killer cells-based immunotherapy for terminal hepatocellular carcinoma patient: a case report

**DOI:** 10.3389/fphar.2023.1203379

**Published:** 2023-08-30

**Authors:** Chan-Ran Park, Hye-Ri Bae, Ga-Young Lee, Chang-Gue Son, Jung-Hyo Cho, Chong-Kwan Cho, Nam-Hun Lee

**Affiliations:** ^1^ Department of Hepatology and Hematology, Graduated School of Korean Medicine, Daejeon University, Daejeon, Republic of Korea; ^2^ East-West Cancer Center, Cheonan Korean Medical Hospital, Daejeon University, Daejeon, Republic of Korea; ^3^ Department of Internal Medicine, Daejeon Korean Medicine Hospital of Daejeon University, Daejeon, Republic of Korea

**Keywords:** case report, cytokine-induced killer cell-based immunotherapy, Gun-Chil-Jung, hepatocellular carcinoma, oncology

## Abstract

**Introduction:** Terminal-stage hepatocellular carcinoma (HCC) is inoperable and currently has no form of adjuvant therapy. This study examined the anticancer herbal extract Gun-Chil-Jung (GCJ) combined with cytokine-induced killer (CIK)-cell-based immunotherapy as a palliative therapy for terminal HCC. We report the case of an HCC patient with extended overall survival and improved symptoms and tumor marker levels following combination therapy with GCJ and CIK cell-based immunotherapy.

**Baseline Characteristics:** From March to July 2020, a 57-year-old man who had been diagnosed with HCC underwent combination treatment with GCJ and CIK cell-based immunotherapy. By August 2021, he was prescribed GCJ. After treatment, the patient’s condition was evaluated with respect to overall survival, tumor markers, symptoms, abdominal computed tomography findings, chest x-ray results, and Eastern Cooperative Oncology Group (ECOG) grade.

**Results:** The patient’s overall survival, tumor marker levels, ECOG grade, and symptoms, including ascites, lower limb edema, jaundice, pleural effusion, and fatigue, were largely alleviated.

**Conclusion:** We expect that this combination therapy may be an option for palliative therapy of terminal HCC.

## 1 Introduction

The most common type of primary liver cancer is hepatocellular carcinoma (HCC), a life-threatening disease with a poor prognosis that most often occurs in patients with chronic liver disease (Craig et al., 2020). Despite the increasing 5-year survival rate in Korea and the recently declining incidence rate of HCC, the majority of patients with advanced-stage HCC suffers from widespread tumor distribution, including extrahepatic spread and vascular invasion, as well as decompensation of liver function (Yu, 2016). Terminal-stage HCC is usually grade 3–4 according to the Eastern Cooperative Oncology Group (ECOG) classification and class C according to the Child-Pugh score; in such cases, only symptomatic treatment is typically recommended (Barone et al., 2013).

An herbal medicine named *Gun-Chil-Jung* (GCJ) is an allergen that removes *Rhus verniciflua* Stokes (RVS) extract. Some studies about the effect of RVS extract on HCC patients have been reported previously [4.5]. There is a case report of a patient with HCC who had no feasible standard management. The progression-free survival (PFS) was over 16 months and 114 months in two advanced HCC patients, respectively, with decreased alpha-fetoprotein (AFP) levels in both after RVS treatment (Chae et al., 2018). A study of the antitumor effects of RVS antitumor effects in tumorigenic hepatocytes of mice showed that it can inhibit tumor cell growth and induce apoptosis (Son et al., 2005).

Cytokine-induced killer (CIK) cell-based immunotherapeutic agent (Immuncell-LC^®^; GC Cell Corp, Seoul, Korea) is an autologous immunotherapy with efficacy that has been reported in several studies (Lee et al., 2015). According to several randomized controlled trials of HCC patients receiving curative treatment, adjuvant CIK cell-based immunotherapy can reduce the recurrence rate of HCC, prevent its metastasis, and prolong overall patient survival, with very few side effects (Takayama et al., 2000; Weng et al., 2008; Hui et al., 2009; Lee et al., 2015).

Therefore, in this study, we examined the combined effect of GCJ and CIK cell-based immunotherapy for palliative care in patients with terminal HCC. In the case presented below, the patient’s symptoms, tumor marker levels, and ECOG grade improved in 17 months of GCJ prescription combined with CIK-cell-based immunotherapy and, as a result, his overall survival was prolonged.

This study followed the Case Report Guidelines with the patient’s informed consent and was approved by the Institutional Review Board of Daejeon University Korean medical hospital (DJUMC-2020-BM-14) (Gagnier et al., 2013).

## 2 Case presentation

### 2.1 Baseline characteristics of the patient

A 57-year-old man with ascites, lower-limb edema, jaundice, pleural effusion, and fatigue was diagnosed with terminal HCC. The disease was diagnosed as Barcelona Clinic Liver Cancer (BCLC) stage D in the BCLC Staging System and presented with poor liver function with a Child-Pugh score of C at Chungnam National University Hospital on March 8. In the absence of curative or adjuvant therapy, the patient visited the Cheonan Korean Medicine Hospital of Daejeon University for a second opinion. He had a history of skin graft surgery in Kangdong Sacred Heart Hospital in 2000, when he got burns in both legs. Furthermore, he was diagnosed with chronic hepatitis C in the same hospital and the same year. He was a non-alcoholic drinker, a non-smoker, and had no hepatitis B. According to him, he never had treatment for hepatitis C. In the computed tomography (CT) scan he brought, taken on March 8, 2020 we found that he has HCC with liver cirrhosis. Based on this history, we assumed that hepatitis C likely resulted in liver cirrhosis and HCC because he did not get proper treatment for hepatitis C. At Chungnam National University Hospital, he was notified that his HCC is cureless. Accordingly, before he visited our hospital, there were no past interventions for his HCC.

From March 20, he was administered GCJ (Supplementary Data S1) twice a day and three cycles of CIK cell-based immunotherapy (April 9 and 27, and July 10). The CIK agent was prepared at a Good Manufacturing Practice facility (GC Cell Corp, Korea) (Supplementary Data S2). Until August 2021, he had steadily received GCJ treatment twice a day. The effects of treatment were assessed based on overall survival (OS), tumor marker level, chest x-ray, abdominal CT images, and ECOG grade.

### 2.2 Overall survival and change of tumor size

**FIGURE 1:**
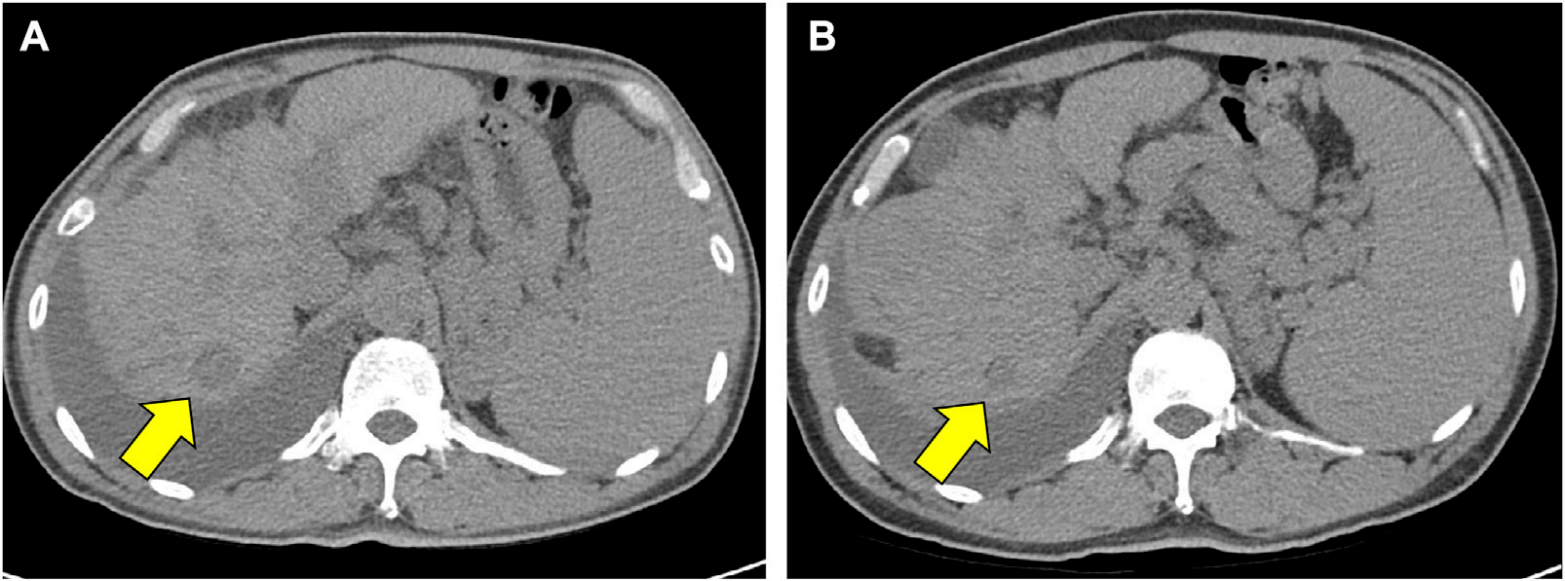
Decrease of tumor size in the CT scan images. **(A)** Is an abdomen CT image which were scanned on July 23 **(B)** is a follow-up abdomen CT image which were scanned on December 22. Yellow arrows indicate a decrease in the longest diameter of tumor mass from 2.28 cm in **(A)**, to 1.60 cm in **(B)**. Abbreviations: CT, computed tomography.

As the patient expired on 15 November 2021, the overall survival was 20.3 months. The exact cause of death is unconfirmed. The survival period based on the last follow-up date (8 September 2021) was 18.3 months. In a follow-up abdominal CT conducted on December 22, there was no clear tumor progression compared with the image from July 23 and the longest diameter of the tumor decreased from 2.28 to 1.60 cm (Figure 1). Thus, the duration of response was 152 days.

### 2.3 Change in the tumor markers and liver function

**FIGURE 2:**
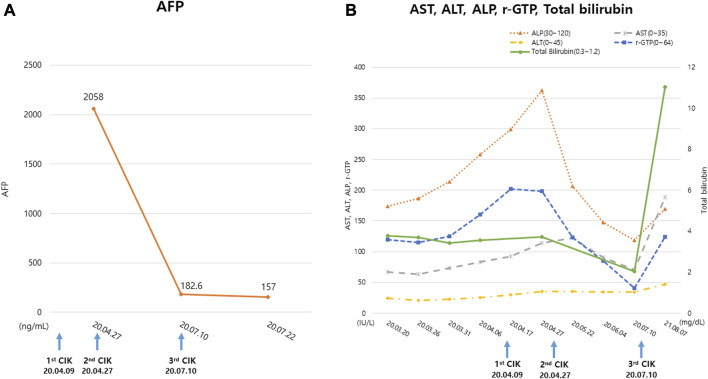
Change in the tumor markers and liver function test. **(A)** The tumor marker, AFP level examined during the treatment period **(B)** The serum ALT, AST, ALP, r-GTP, Total bilirubin levels examined during the treatment period. The blue arrows indicate the date of CIK cell-based immunotherapy administration. ‘CIK’ means CIK cell-based immunotherapy. Abbreviations: AFP, alpha-fetoprotein; ALP, alkaline phosphatase; ALT, alanine aminotransferase; AST, aspartate aminotransferase; CIK, Cytokine-induced killer; r-GTP, gamma glutamyl transpeptidase.

The AFP level of the patient on April 27 was 2,058 ng/mL but decreased to 157 ng/mL on July 22. His serum levels of gamma-glutamyltransferase, alkaline phosphatase, and total bilirubin decreased during treatment while his alanine aminotransferase and aspartate aminotransferase levels remained unchanged. During combined GCJ prescription and CIK cell-based immunotherapy treatment, the liver function showed an improved tendency. Meanwhile, roughly a year after the last cycle of CIK cell-based immunotherapy, the overall liver function test levels increased again (Figure 2).

### 2.4 Ascites and pleural effusion

**FIGURE 3:**
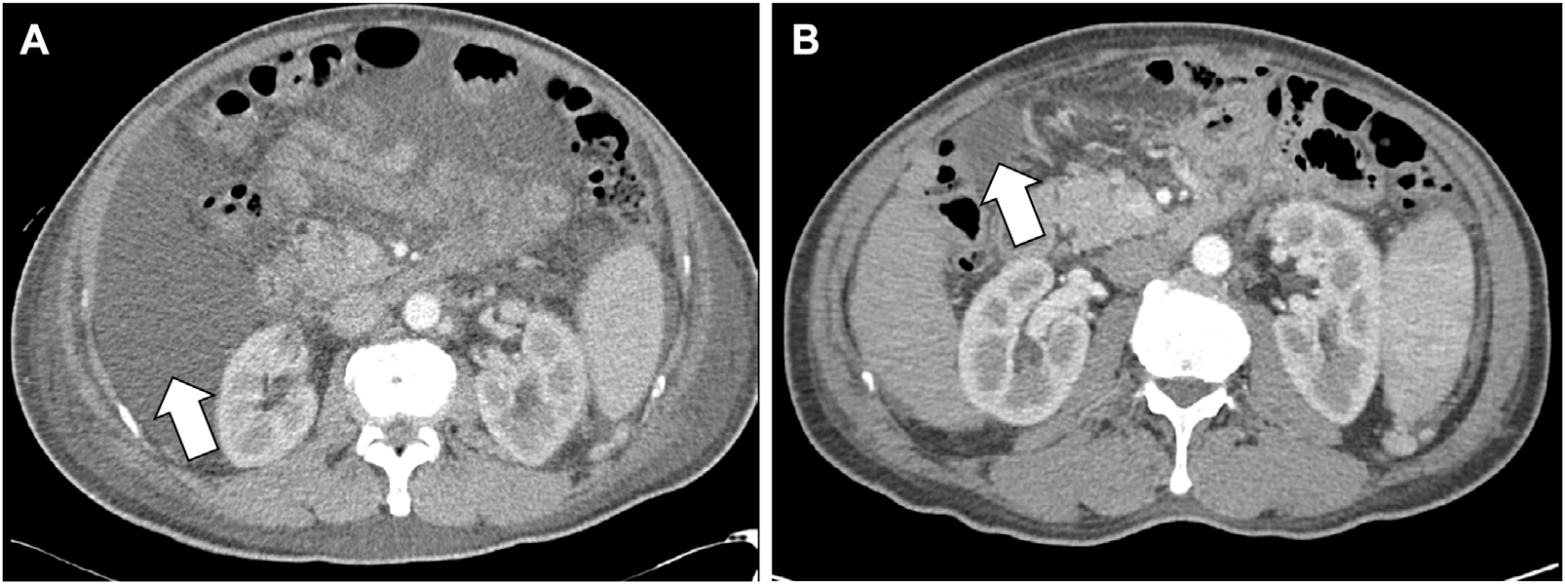
Decrease of ascites in the CT scan images. **(A)** Is an abdomen CT image which were scanned on March 8 **(B)** is a follow-up abdomen CT image which were scanned on July 23. White arrows indicate a shrinkage of ascites in **(B)**, compared to **(A)**. Abbreviations: CT, computed tomography.

**FIGURE 4:**
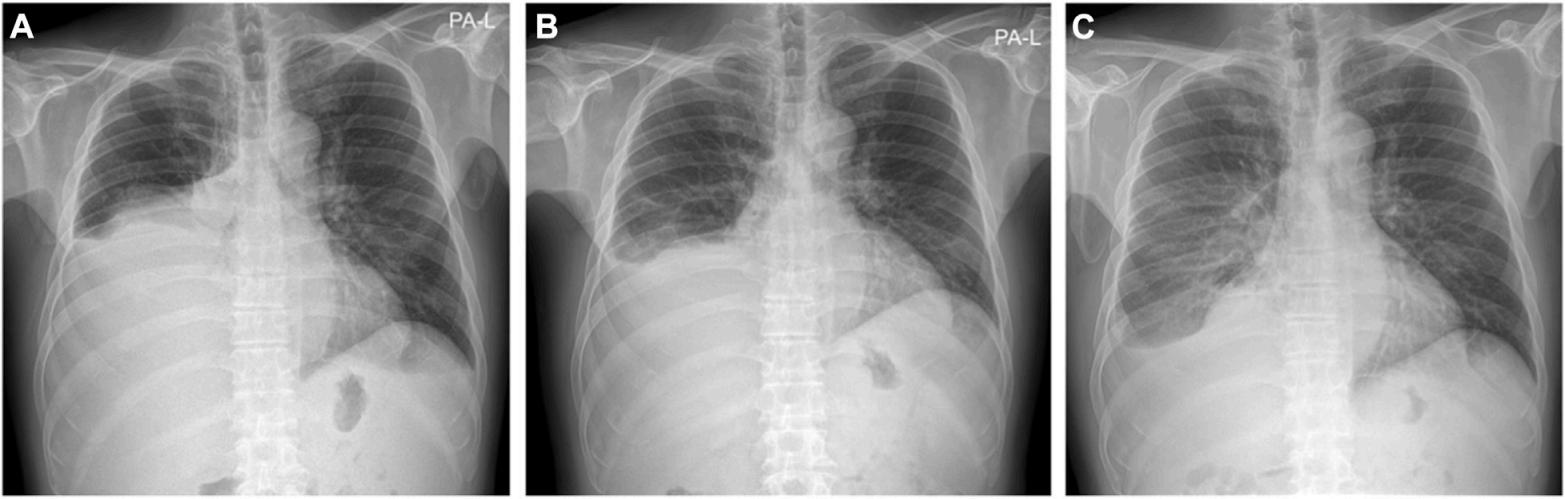
Chest X-ray images. **(A)** Chest X-ray on July 10 **(B)** Chest X-ray on October 23 **(C)** Chest X-ray on December 10. Even after he stopped taking diuretic from July 27, the chest X-ray findings above show decrease of pleural effusion.

Because of ascites and pleural effusion, prior to combination therapy, the patient frequently underwent paracentesis, despite taking a regularly prescribed diuretic. After combination therapy, the ascites accumulated more slowly, resulting in a decrease in the patient’s abdominal circumference from 96 to 87 cm. Abdominal CT conducted on July 23 indicated ascites shrinkage compared to the image obtained on March 8 (Figure 3). Even after the patient stopped taking diuretics from July 27, follow-up chest x-rays conducted on October 23 and December 10 showed a reduction in pleural effusion compared with the image from July 10 (Figure 4). The patient was still alive and relatively healthy without needing a paracentesis or diuretics, until August 2021, approximately 3 months before he expired.

### 2.5 Physical performance

Based on these changes, the patient’s ECOG grade decreased from 3 to 2. He was able to engage in social activities and both his physical strength and appetite improved.

### 2.6 Adverse event and safety

During the treatment process, no serious adverse events occurred. Chemotherapy or small-molecule inhibitors usually cause adverse events such as fatigue, diarrhea, and reduced quality of life (Kamal et al., 2022). Compared with standard treatment options for advanced HCC, GCJ combined with CIK cell-based immunotherapy has safety benefits.

## 3 Discussion

Despite the increase in first-line treatment options including Atezolizumab–Bevacizumab, Sorafenib, and Lenvatinib, terminal HCC treatment cannot cure HCC. It aims to control the cancer, relieve its symptoms, and give patients a good quality of life. In a meta-analysis, the survival of patients with terminal HCC was estimated at a 1-year survival rate of 11% (95% CI, 4.7–22; range, 0%–57%) (Cabibbo et al., 2010). Terminal HCC should thus be managed with palliative support, including nutritional supplements, pain control, and psychological assistance (Kumar and Panda, 2014).

While immunotherapies have made great strides in the fight against HCC, single immunotherapy has shown that a high percentage of patients still fail to respond and that tumors have the potential to become resistant to terminal HCC. Therefore, this study focused on the potential of combining GCJ with CIK-cell-based immunotherapy to improve the response rates and long-term outcomes of patients.

Our patient had terminal HCC, for which he received palliative care with GCJ and CIK cell-based immunotherapy. The efficacy of these two therapies for HCC has been reported in previous studies (Takayama et al., 2000; Wang et al., 2002; Son et al., 2005; Weng et al., 2008; Hui et al., 2009; Kim et al., 2010; Lee et al., 2015; Chae et al., 2018). RVS extract is an anticancer substance that promotes cancer cell apoptosis, suppresses cancer cell growth, and inhibits angiogenesis (Choi et al., 2012). Some studies have demonstrated its effect on various kinds of cancers. For example, in a clinical study with 40 non-small cell lung cancer (NSCLC) patients, oral administration of RVS extract prolonged OS and PFS rate (Cheon et al., 2011). In a case study about a gastric cancer patient, the tumor shrank after 5 months of treatment with orally administered RVS extract (Lee et al., 2010). Moreover, an *in vitro* study using biliary tract cancer cells shows that RVS extract downregulates the proliferation and upregulates the apoptosis of cancer cells (Joung and Kim, 2015). Another *in vitro* study with breast cancer cells demonstrated that RVS treatment induces cancer apoptosis through the Adenosine monophosphate (AMP)-activated protein kinase signaling pathway (Lee et al., 2014). The main compounds of GCJ, an herbal extract of RVS, are fisetin, fustin, and sulfuretin, all of which have apoptotic actions in diverse types of cancer (Moon et al., 2015; Jun et al., 2020). Fisetin is an apoptotic component for prostate, pancreatic, and colon cancer cells (Khan et al., 2008; Suh et al., 2008; Murtaza et al., 2009). Sulfuretin also induces apoptosis in leukemia cells through the Fas-mediated caspase-8–dependent pathway, which activates apoptotic factors (Lee et al., 2012). GCJ has traditionally been used to relieve blood stasis and promote detoxification (Yoo and Roh, 1977). The benefits of RVS extract in a patient with post-liver transplantation recurrent HCC and lung metastasis have been described in a case study (Kim et al., 2010). The positive effects include prolonged survival and the shrinkage of the metastatic region of the lung (Kim et al., 2010).

CIK cell-based immunotherapy consists of a mixture of T lymphocytes comprising CD3^+^/CD56^+^ cells, CD3^+^/CD56^-^ cytotoxic T cells, and CD3^-^/CD56^+^ natural killer cells. The mixture was prepared from the patient’s peripheral blood, and mononuclear cells inside the blood were cultured *ex vivo* through co-stimulation with the anti-CD3 antibody and interleukin-2 (Lee et al., 2015). The antitumor cytotoxic activity and tumor growth inhibition of CIK cells in HCC have been examined both *in vitro* and *in vivo* (Wang et al., 2002). CIK cells are estimated to be involved in eliminating HCC cells, likely through interactions with leukocyte function-associated antigen-1, which is related to cytolysis in HCC target cells (Wang et al., 2002). Moreover, significantly improved OS and recurrence- or progression-free survival were shown in numerous trials including advanced HCC (Zhang and Schmidt-Wolf, 2020; Han et al., 2022).

The combined use of GCJ and CIK cell-based immunotherapy may improve immune function in tumors. The mild adverse events and multiple improvements in their anti-cancer activity make GCJ and CIK-cell-based immunotherapy a favorable therapeutic option in cancer immunotherapy. Combining herbal medicine and adoptive cell therapy decreased tumor markers, such as alpha-fetoprotein (AFP), and improved immune functions, such as those involving CD3^+^, while increasing CD3^+^CD56^+^ and CD3^+^CD8^+^ cell ratios in the peripheral blood. This indicates that continued decreases in AFP concentrations after CIK cell therapy may be the pathway via which CIK and GCJ exert their roles in preventing short-term progression, which can be used to predict the clinical efficacy of CIK-based immunotherapy as a form of maintenance treatment for patients with terminal HCC. In this study, in approximately 17 months of GCJ treatment combined with CIK cell-based immunotherapy, the patient’s outcome was favorable, as indicated by better performance status and decreased ascites, pleural effusion, and tumor marker levels. Liver function levels improved when CIK cell-based immunotherapy was combined with GCJ, although it aggravated after the last cycle of the therapy ended. This indicates that GCJ treatment may be more effective when combined with CIK cell-based immunotherapy.

As a result, the patient’s overall survival extended to 20.3 months. This is an encouraging outcome compared to the median survival period of terminal HCC patients of three to four months (Cabibbo et al., 2010). This suggests that GCJ is a promising candidate for anti-cancer drugs with a gamut of therapeutic applications.

The limitations of this case are as follows: First, although this case has achieved long-term response duration, it is not universally representative. Second, the combined effect requires further clarification. Third, the patient was followed up retrospectively, and the pharmacokinetic/pharmacodynamic activity of T cells could not be accurately detected. With further research, the mechanism of combination immunotherapy should be further explored.

## 4 Conclusion

This case report demonstrates the utility of combination treatment with GCJ and CIK cell-based immunotherapy to extend OS and improve tumor marker levels, tumor-related symptoms, and ECOG grade in a patient with terminal HCC. This study reported a favorable therapeutic effect on patients; immunotherapy may be a potentially feasible systemic treatment for terminal cancers that cannot be cured or treated. Clinical trials and systematic studies with a sufficient number of patients are needed to further corroborate these results.

## Data Availability

The original contributions presented in the study are included in the article/Supplementary Material, further inquiries can be directed to the corresponding author.

## Supplementary material

SUPPLEMENTARY FIGURE S1 Fingerprinting analysis of GCJ components. **(A)** Fingerprinting analysis of standard solution: **(B)** Fingerprinting analysis of GCJ: **(C)** Contents of GCJ components. Abbreviations: GCJ, Gun-Chil-Jung.
