# Supplementary material for: Feasibility of combination of Gun-Chil-Jung and cytokine-induced killer cells-based immunotherapy for terminal hepatocellular carcinoma patient: a case report
Source: Front Pharmacol. 2023 Aug 30;14:1203379. doi: 10.3389/fphar.2023.1203379 (PMC10502300; doi:10.3389/fphar.2023.1203379)
Supplement: Supplementary file 1 [file Table1.DOCX]

Supplementary data 2. Summary of Injected CIK Agents

|  | **1^st^ CIK agent** | **2^nd^ CIK agent** | **3^rd^ CIK agent** |
| --- | --- | --- | --- |
| **Total Cell Count** | 4.5 x 10^9^ cells | 3.9 x 10^9^ cells | 4.6 x 10^9^ cells |
| **Cell viability** | 96.4% | 97.5% | 91.3% |
| **CD3+ cell** | 99.3% | 99.8% | 99.6% |
| **CD8+ cell** | 77.8% | 93.0% | 90.0% |
| **CD56+ cell** | 10.1% | 25.9% | 17.2% |
| **CD14+ cell** | 0.1% | 0.0% | 0.0% |
| **CD20+ cell** | 0.2% | 0.1% | 0.5% |
