# Supplementary figures and images for: Feasibility of combination of Gun-Chil-Jung and cytokine-induced killer cells-based immunotherapy for terminal hepatocellular carcinoma patient: a case report
Source: Front Pharmacol. 2023 Aug 30;14:1203379. doi: 10.3389/fphar.2023.1203379 (PMC10502300; doi:10.3389/fphar.2023.1203379)

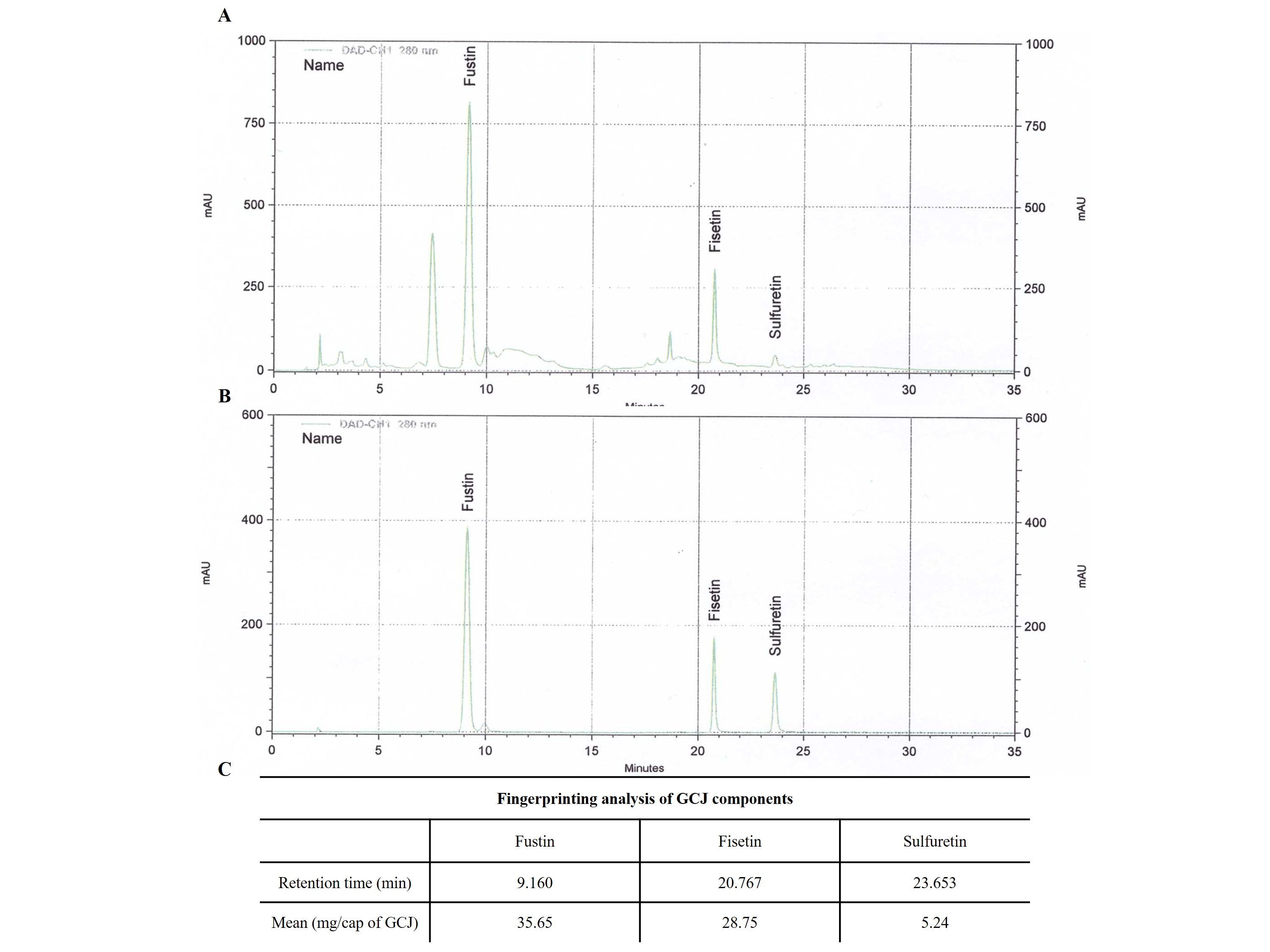

Supplement: Supplementary file 2 [file Image1.TIF]
